# Incidence and survival of childhood central nervous system tumors in Denmark, 1997–2019

**DOI:** 10.1002/cam4.4429

**Published:** 2021-11-19

**Authors:** Anne Sophie Lind Helligsoe, Line Kenborg, Louise Tram Henriksen, Aparna Udupi, Henrik Hasle, Jeanette Falck Winther

**Affiliations:** ^1^ Department of Pediatrics and Adolescent Medicine Aarhus University Hospital Aarhus Denmark; ^2^ Department of Clinical Medicine Faculty of Health Aarhus University Aarhus Denmark; ^3^ Childhood Cancer Research Group Danish Cancer Society Research Center Copenhagen Denmark; ^4^ Biostatistical Advisory Service (BIAS) Faculty of Health Aarhus University Denmark

**Keywords:** central nervous system tumor, childhood cancer, childhood cancer survivors, incidence, survival

## Abstract

**Background:**

Incidence rates in Denmark of central nervous system (CNS) tumors remain among the highest in the world. Survival rates, however, have improved in the past decades in high‐income countries.

**Methods:**

We analyzed incidence and survival of childhood CNS tumors in Denmark diagnosed from 1997 to 2019 based on data from the Danish Childhood Cancer Registry and information on histological types, tumor localization, and treatment from medical records.

**Results:**

From 1997 to 2019, 949 children<15 years were diagnosed with a CNS tumor. Age‐standardized incidence was 42.1 (95% CI, 39.4–44.6) per million person‐years and stable during this period. Age‐specific incidence for children aged 0–4 years was 47.7 per million. More than one‐third (*n *= 374, 39.4%) were treated with surgery alone. Overall survival rates 5 and 10 years after diagnosis were 77.6% (95% CI, 74.7–80.2) and 74.7% (95% CI, 71.7–77.5). Five‐year overall survival improved from 73.0% (95% CI, 68.9–76.7) in 1997–2008 to 83.2% (95% CI, 79.2–86.4) in 2009–2019 (*p*‐value < 0.0001) in children aged 0–4 years (*p *= 0.0006).

**Conclusion:**

Incidence rates are stable but remain among the highest in the world. Despite improved survival rates in recent years in younger children, some subtypes still have a poor prognosis.

## Graphical abstract

Incidence of childhood central nervous system (CNS) tumors in Denmark from 1997 to 2019 was 42.1 per million person‐years and did not increase from 1997 to 2019. Survival has improved especially among younger children with CNS tumors. Still, some subgroups in particular have a poor prognosis.

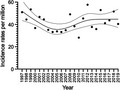

## 1 INTRODUCTION

Although cancer in children is rare, it is the second leading cause of death among children in high‐income countries.^1^ Central nervous system (CNS) tumors are the most common solid malignancy in childhood, representing 20% of all cancers observed in children.^2^ Furthermore, the mortality rate is high. Thus, CNS tumors contribute considerably to the overall childhood cancer incidence and mortality of childhood cancer. A study of the international incidence of childhood cancer between 2001 and 2010 reported an increase in the global incidence of childhood cancer, including CNS tumors.^3^ However, the incidence of CNS tumors varied across populations and appeared to be highest (>20 per million person‐years) in high‐income countries including the United States and the Nordic countries,^4^, ^5^, ^6^, ^7^ and lowest in low‐ and middle‐income countries.^7^ Whether the high incidence can be explained by biological factors, the availability of better diagnostic facilities, and/or more complete registration of cancer diagnoses in national registries remains uncertain.^8^, ^9^, ^10^ Since 1943, data on the incidence of cancer in the Danish population have been registered in the Danish Cancer Registry. Using these complete and comprehensive Danish data, a recently published study^10^ reported an increase in childhood CNS tumors, with an age‐standardized rate reaching 43.8 per million person‐years in 1977–2014, but the study lacked detailed information on histological types and treatment. In Europe, annual incidence rates range from 22 to 44 per million person‐years and increased from the 1980s to the 1990s,^5^, ^8^, ^11^ with a decline or stabilization in the years after 2000.^2^, ^3^, ^6^, ^12^, ^13^

Historically, survival of pediatric CNS tumors has improved in Denmark, from a 5‐year survival rate of 66% in the 1980s to stable rates of 70%–80% from 2000 to 2020 according to WHO databases.^14^, ^15^

Changes in incidence and survival are caused by numerous factors such as access to health care and improved diagnostic and treatment facilities,^3^ and studies reporting the development over time can form the basis for etiological research and monitor the progress in treatment of childhood cancer. As CNS tumors are a heterogeneous group of malignancies with varied incidences and survivals, detailed tumor information is crucial in these types of studies. Thus, in the present study, we analyzed incidence and survival in children with CNS tumors in Denmark diagnosed, 1997–2019, according to histological type and tumor localization. We also reported the treatment modalities of distinct CNS tumors that have not previously been reported in Danish children in incidence and survival studies.

## 2 MATERIALS AND METHODS

### 2.1 Patient cohort

Since 1985, all children diagnosed with cancer in Denmark have been registered by clinicians in the nationwide clinical database, the Danish Childhood Cancer Registry (DCCR), with the overall aim of monitoring the quality of childhood cancer care in Denmark.^16^ The DCCR is linked to the Danish National Patient Registry, the National Pathology Registry, and the Danish Cancer Registry using the unique national identification number assigned to all permanent residents in Denmark at birth or immigration. We obtained information on all children below 15 years of age, living in Denmark who had a primary CNS tumor diagnosed from 1 January 1997 to 31 December 2019 (*n* = 1093), including information on known syndromes predisposing to CNS tumors (neurofibromatosis type 1 or 2, tuberous sclerosis, Gorlin–Goltz, or multiple endocrine neoplasia type 1), histological type, treatment for first primary tumor, and survival. All patients with neurofibromatosis type 1 or 2 and low‐grade glioma as well as patients with tuberous sclerosis and subependymal giant cell astrocytoma were registered. Children with other tumors in the CNS such as Langerhans cell histiocytosis, lipoma, lymphoma, hemangioma, hamartoma, sarcoma, and bone sarcoma were excluded (*n* = 144), as they are not classified as classical CNS tumors. Information on children aged 15–18 years was not complete for the entire study period and was therefore not included. The final patient cohort consisted of 949 children.

To describe overall and specific treatment modalities, we validated all information on tumor localization and treatment characteristics obtained from the DCCR. Missing information was obtained by systematically reviewing and abstracting relevant information from all medical reports except 62 (6.5%), which were not available.

### 2.2 Tumor classification

Tumors were classified according to localization: cerebellum, cerebrum, supratentorial central area, brain stem, hypothalamic and pituitary area, optic nerve or chiasma, pineal gland, or intraspinal. Both benign and malignant tumors were classified histologically on the basis of the International Classification of Diseases for Oncology (ICD‐O)^17^ according to main diagnostic group *III CNS and miscellaneous intracranial and intraspinal neoplasms* and the subgroup *Xa Intracranial and intraspinal germ cell tumors* in the International Classification of Childhood Cancer, third edition (ICCC‐3) (Table S1).^7^ According to 2016 CNS WHO,^18^ we adopted the terminology “embryonal CNS Tumor NOS” to replace primitive neuroectodermal tumor (CNS‐PNET). Atypical teratoid/rhabdoid tumor (ATRT) was incorporated in the ICD‐O as a new entity in 2000. Optic nerve glioma and brain stem glioma were accepted without biopsy due to characteristic presentation.

### 2.3 Statistical methods

#### 2.3.1 Incidence

The overall age‐specific incidence rates (ASI) per million person‐years were calculated for all types of CNS tumors in the three age groups 0–4 years, 5–9 years, and 10–14 years according to date of birth and calendar year, with the annual Danish population at midyear from Statistics Denmark^19^ as the denominator. ASI was calculated for specific histological types and localization in children aged 0–4 years, 5–9 years, and 10–14 years and separately for boys and girls.

Age‐standardized incidence rates (ASRs) expressed per million person‐years were derived by direct standardization, using the weight of the world standard population as proposed by Segi^20^ in order to compare with other countries and to eliminate changes in the age distribution during the study period. ASRs were calculated overall and for each year.

Finally, we evaluated changes in incidence rate over time by analyzing the age‐standardized incidence rates using restricted cubic spline smoother for the year of diagnosis with four knots, in a Poisson regression model. A joint post hoc test of the coefficients of the higher order spline variables gave a formal test for nonlinear trend.

#### 2.3.2 Survival

Survival was defined as time from diagnosis to death from any cause and presented with median with lower and upper quartiles. Patients were censored at date of emigration or 31 December 2019, whatever occurred first. We calculated both 5‐ and 10‐year overall survival (OS) for all histological types combined and for specific subgroups for the entire study period and for the two time periods (1997–2008 and 2009–2019) separately. All survival rates were reported with 95% confidence intervals (95% CIs). Survival function was estimated by the Kaplan–Meier method and presented in plots in children aged 0–4 years and 5–14 years to facilitate comparison with previous studies. The difference in survival functions was estimated with the log‐rank test. Two children died at birth. To include these two cases in the survival analyses, 1 day was added to all cases.

Survival function and 95% CIs for each gender, age group, time period, and localization were calculated for individual tumor types. To identify possible factors influencing survival, the risk of death according to gender, age group, time period, and localization was estimated by hazard ratios (HRs) and their 95% CIs using univariate and multivariate Cox proportional hazards regression models. Reference categories were selected logically as the first category (boys, children aged 0–4 years, supratentorial location, 1998–2008).

## 3 RESULTS

### 3.1 Patient cohort

After exclusion of the 144 non‐CNS tumors, we identified 949 children (468 girls, 49.3%) diagnosed with a CNS tumor in Denmark who were younger than 15 years of age at diagnosis. On average, 41.3 new cases were diagnosed annually (Table 1). Children aged 0–4 years accounted for 38.2% of all the cases, followed by children aged 5–9 years (33.7%) and children aged 10–14 years (28%). The median follow‐up time was 7.5 years (quartiles 1.8–14.9). Around 7% (*n *= 69) had a predisposing condition: 55 children (5.8%) were diagnosed with neurofibromatosis 1 or 2, 10 (1.1%) with tuberous sclerosis, and 4 (0.4%) with either Gorlin–Goltz syndrome or multiple endocrine neoplasia type 1.

**TABLE 1:** Characteristics of children <15 years diagnosed with a CNS tumor in Denmark, 1997–2019

	*n*	%
Patients	949	100
Gender		
Girls	468	49.3
Boys	481	50.7
Median follow‐up (interquartile range)	7.5	(1.8–14.9)
Year of diagnosis		
1997–2005	377	39.7
2006–2013	319	33.6
2014–2019	263	27.7
Age at cancer diagnosis		
0–4	363	38.2
5–9	320	33.7
10–14	266	28.0
Location of tumor		
Cerebellum	342	36.0
Cerebrum	179	18.9
Supratentorial central area	89	9.4
Hypothalamus or pituitary region	84	8.9
Brain stem	73	7.7
Optic nerve or chiasma	65	6.8
Intraspinal	45	4.7
Pineal gland	22	2.3
Unknown	50	5.3
Predisposing syndrome		
Neurofibromatosis type 1 or 2	55	5.8
Tuberous sclerosis	10	1.1
Gorlin–Goltz or multiple endocrine neoplasia type 1	4	0.4
Cancer treatment		
Surgery (only)	374	39.4
Chemotherapy	328	34.6
Radiation	293	30.9
No treatment	93	9.7
No treatment information	41	4.3
Surgery	748	
Macroscopically complete	418	55.9
Partial resection	211	28.2
Biopsy only	86	11.5
Extent unknown	33	4.4

### 3.2 Treatment characteristics

In total, 374 (39%) children were treated only surgically and 93 patients were observed without therapy (Table 1). Surgery, either alone or combined with radiation or chemotherapy, was performed in 748 children (79%) ranging from macroscopically complete resection (*n* = 418) to partial resection (*n* = 211) and biopsy (*n *= 86). In 33 (4.4%) children undergoing surgery, the extent of surgery was unknown. Chemotherapy was administered in 328 (35%) children and radiotherapy in 293 (31%) children. Treatment with radiotherapy was equally distributed in the two time periods: 12.6 children per year received radiotherapy in 1997–2008 (30.5% of children diagnosed) compared to 12.9 children per year from 2009 to 2019 (31.2% of children diagnosed). More children were treated with chemotherapy in 2009–2019 (183/494, 37.0% of children diagnosed) compared to 1997–2008 (145/455, 31.9%). Information about radiation fields was not available.

### 3.3 Tumor localization and subgroups according to ICCC‐3

The most predominant tumor location was the cerebellum (*n* = 342, 36%) followed by the cerebrum (*n* = 179, 19%), supratentorial central area (*n* = 89, 9.4%), hypothalamus or pituitary region (*n* = 84, 8.9%), brain stem (*n* = 73, 7.7%), optic nerve or chiasma (*n* = 65, 6.8%), intraspinal (*n* = 45, 4.7%), and pineal gland (*n* = 22, 2.3%) (Table 1). The most common histological types were astrocytoma (*n* = 385, 40.6%) followed by embryonal tumors (*n* = 160, 16.9%) (Table 2). In 171 cases (18%), the diagnoses were not verified histologically, because it was not necessary to establish the diagnosis or the tumor was surgically inaccessible (data not shown).

**TABLE 2:** Subgroups according to the International Classification of Childhood Cancer, third edition (ICCC‐3) of all CNS tumors diagnosed in Denmark in children <15 years from 1997 to 2019 presented with median age, gender distribution, and treatment information

	*n*	% of CNS tumors	Age at diagnosis, median (range)	Gender distribution, Girls, *n*	%	Surgery (*n*)	Chemotherapy (*n*)	Radiation (*n*)
All CNS tumors	949	100	7.0 (0–15)	468	49.3	748	328	293
IIIa. Ependymomas and choroid plexus tumor	77	8.1	3.0 (0.1–14.9)	35	45.5	73	31	38
IIIa1. Ependymoma	56	—	5.1 (0.1–14.9)	21	37.5	53	25	35
IIIa2. Choroid plexus tumor	21	—	1.0 (0.2–10.6)	14	66.7	20	6	3
IIIb. Astrocytomas	385	40.6	6.7 (0–14.9)	21	55.6	309	96	62
IIIb1. Pilocytic	200	—	7.1 (0.1–14.7)	111	55.5	192	31	17
IIIb2. Optic glioma	50	—	3.8 (0.4–12.1)	33	66.0	11	23	6
IIIb3. Glioblastoma	23	—	8.0 (0.9–14.5)	12	52.2	20	16	14
IIIb4. Anaplastic	17	—	8.0 (3.0–14.7)	10	58.9	16	9	12
IIIb5. Diffuse	15	—	9.2 (1.0–13.8)	8	53.3	14	5	4
IIIb6. Subependymal giant cell	12	—	9.9 (0.4–14.9)	5	41.7	11	<3	0
IIIb7. Pleomorphic xanthoastrocytoma	9	—	10.3 (3.7–13.5)	7	77.8	8	<3	<3
IIIb8. Pilomyxoid	4	—	9.8 (2.9–14.1)	2	50.0	4	<3	0
IIIb9. Fibrillary	4	—	2.7 (0.74–13.3)	3	75.0	4	<3	0
Other	51	—						
IIIc. Intracranial and intraspinal embryonal tumors	160	16.9	5.7 (0–14.3)	72	45.0	150	130	113
IIIc1. Medulloblastoma	124	—	5.9 (0.1–14.3)	50	40.3	118	105	91
IIIc2. Embryonal CNS tumor NOS	16	—	7.1 (0.6–12.3)	8	50.0	15	10	9
IIIc3. Medulloepithelioma	<6	—	—	—	—	—	—	—
IIIc4. Atypical teratoid/rhabdoid tumor	19	—	1.3 (0–11.5)	14	73.7	16	15	13
IIId. Other gliomas	66	7.0	6.9 (0.6–15.0)	31	47.0	33	24	26
IIId1. Oligodendroglioma	13	—	9.4 (0.6–15.0)	7	53.9	12	3	3
IIId2. Mixed and unspecified glioma	53	—	6.2 (1.1–14.8)	24	45.3	21	23	23
IIIe. Other specified intracranial and intraspinal neoplasms	152	16.0	8.8 (0–15)	67	44.1	129	15	24
IIIe1. Pituitary adenoma and carcinoma	25	—	10.2 (0.9–14.8)	9	36.0	15	<3	2
IIIe2. Craniopharyngioma	35	—	7.4 (0–14.5)	16	45.7	34	0	6
IIIe3. Pineal parenchymal tumor	11	—	11.1 (0.9–15.0)	7	63.6	4	3	5
IIIe4. Mixed glial‐neuronal tumor	40	—	9.6 (0–14.4)	20	50.0	38	9	8
IIIe5. Dysembryoblastic neuroepithelial tumor (DNET)	18	—	10.1 (0.4–14.3)	3	16.7	17	<3	<3
IIIe6. Ganglioglioma	17	—	8.8 (0.6–14.7)	10	58.8	15	0	<3
IIIe7. Schwannoma	<6	—	8.1 (1.4–14.9)	2	33.3	6	<3	0
IIIf. Unspecified intracranial and intraspinal neoplasms	15	1.6	8.7 (0.0–13.5)	5	33.3	14	<3	<3
IIIf1. Meningioma	15	—	8.7 (0–13.5)	5	33.3	14	<3	<3
Xa. Intracranial and intraspinal germ cell tumors	32	3.4	9.8 (0.0–14.7)	16	50.0	28	22	21
Xa1. Germinoma	18	—	10.4 (13.9–14.7)	8	44.4	15	17	17
Xa2. Non‐germinoma germ cell tumors	14	—	3.7 (0–14.7)	8	57.1	13	4	4
Unclassified	62	6.5	6.5 (0.0–14.1)	28	45.2	12	8	7

Medulloblastoma (*n* = 124, 50 girls) was diagnosed predominantly in children aged 5–9 years (*n* = 54), followed by children aged 0–4 years (*n* = 46) and less frequently at 10–14 years (*n* = 24) (Table S2). ATRT (*n* = 19, 14 girls) was diagnosed primarily in the very young children. The majority of these children received both chemotherapy (*n* = 16) and radiation (*n* = 13), two received only radiation, and treatment information was unavailable in two children.

Looking further into tumors in children <24 months at diagnosis (*n* = 147, 76 girls), the three most dominant histological types were astrocytoma (*n* = 48), including 21 with pilocytic astrocytoma, medulloblastoma (*n* = 16), and ATRT (*n* = 16) (Table S2).

The three most dominant histological types among children with tumors with an intraspinal location (*n* = 45, 22 girls) were astrocytoma (*n* = 15), other specified intraspinal neoplasm (*n* = 15), and ependymoma (*n* = 9) (Table S4).

### 3.4 Age‐standardized and age‐specific incidences

**FIGURE 1:**
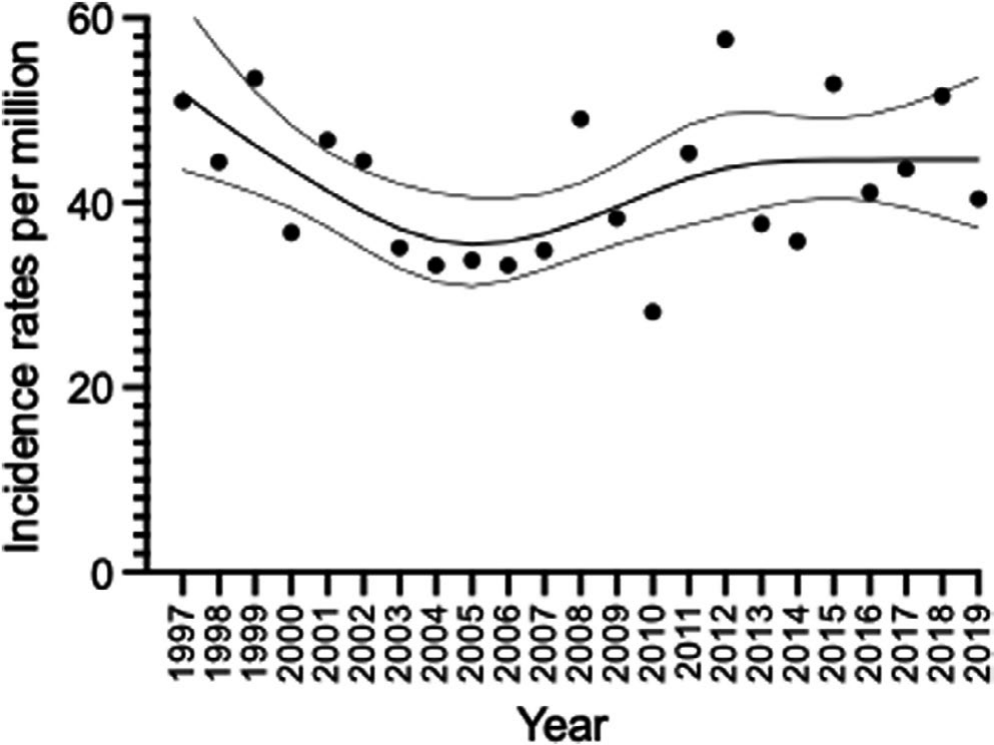
Age‐standardized incidence rates (dots) with a smoothing spline with 95% CI (grey lines) of childhood CNS tumors in Denmark diagnosed from 1997–2019 in children aged 0–14 years including all subgroups as classified as CNS in ICCC‐3

The overall ASR for the entire study period was 42.1 (95% CI 39.6–44.9, Figure 1). The overall ASR for brain tumors only was 40.1 (95% CI 37.6–42.8) per million person‐years.

ASR was clinically stable from 1997 to 2019 within the range of 31.0–61.7 although the statistical test indicated evidence for nonlinear relation between log of the incidence and calendar year (*p* = 0.0071).

ASIs of all CNS tumors in the three age groups are shown in Table S3. The highest ASI was observed in children aged 0–4 years, with 47.7 per million person‐years, followed by 40.7 in children aged 5–9 years and 34.7 in children aged 10–14 years (Figure S1).

### 3.5 Incidence according to ICCC‐3 subgroups

Astrocytomas (*n* = 385, 214 girls) were the most predominant histological type in children aged 0–4 years, with an ASI of 20.7 per million person‐years (Table S3) followed by 16.8 in children aged 5–9 years and 13.4 in children aged 10–14 years. Age distribution and treatment characteristics for the three major sub‐types of astrocytomas and other specific important subgroups are given in Table S2.

### 3.6 Survival

**FIGURE 2:**
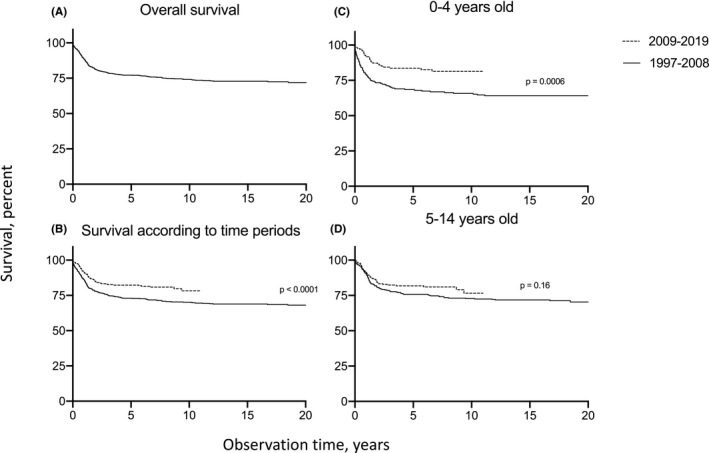
Survival of CNS tumors in Denmark in children 0–14 years old. (A) Overall survival from 1997–2019 (B) Survival in children diagnosed from 1997–2008 compared with 2009–2019 (*p *< 0.0001) (C) Survival in children aged 0–4 years diagnosed in 1997–2008 or 2009–2019 (*p* = 0.0006) (D) Survival in children aged 5–14 years diagnosed in 1997–2008 or 2008–2019 (*p *= 0.16)

At the end of the period, 232 (24.5%) had died, with a median time after diagnosis of 1.07 years (quartiles 0.53–2.1). Five‐year OS for all tumors combined was 77.6% (95% CI 74.7–80.2) (Table 3) and 74.7% (95% CI 71.7–77.5) at 10 years (Figure 2). Five‐year OS improved from 73.0% (95% CI 68.9–76.7) in 1997–2008 to 83.2% (95% CI 79.2–86.4) in 2009–2019 (*p* = <0.0001) (Figure 2).

**TABLE 3:** Five‐year overall survival among children with CNS tumors diagnosed between 1997 and 2019 according to ICCC‐3 subgroups, by gender, age at diagnosis, localization, and time period

	III‐CNS 5‐y OS (95% CI)	IIIa‐Ependymomas and choroid plexus tumors 5‐y OS (95% CI)	IIIb‐Astrocytomas 5‐y OS (95% CI)	IIIc‐Embryonal tumors 5‐y OS (95% CI)	IIId‐Other gliomas 5‐y OS (95% CI)	IIIe‐Other specified tumors 5‐y OS (95% CI)	IIIf‐Unspecified 5‐y OS (95% CI)	Xa‐Germ cell tumors 5‐y OS (95% CI)	Unclassified
	77.6 (74.7–80.2)	69.8 (57.4–79.2)	83.9 (79.8–87.3)	59.3 (50.9–66.7)	61.3 (47.6–72.4)	90.9 (84.9–94.6)	86.7 (56.4–96.5)	93.2 (75.1–98.3)	64.1 (50.4–75.0)
Gender									
Boys	78.0 (73.8–81.6)	72.0 (54.0–84.0)	83.1(76.4–88.0)	59.2 (47.8–69.0)	72.3 (52.1–85.1)	93.0 (84.2–96.6)	80.0 (40.9–94.6)	100	65.5 (46.3–79.2)
Girls	76.7 (72.5–80.4)	67.0 (48.3–80.2)	84.6 (78.9–88.9)	59.4(46.6–70.2)	50.4 (31.6–66.5)	88.7(77.7–94.4)	100	87.1 (57.3–96.6)	62.2 (41.1–77.6)
Age at diagnosis									
0–4 years	76.1 (71.2–80.3)	66.6 (49.9–78.8)	90.0 (83.9–93.8)	54.7 (41.7–66.0)	77.3 (50.1–90.8)	84.8 (67.2–93.4)	75.0 (12.8–96.1)	88.9 (43.3–98.4)	50.4 (30.3–67.5)
5–14 years	78.1 (74.4–81.4)	74.5 (0.54–0.87)	80.0 (74.1–84.6)	62.6 (51.4–72.0)	54.6 (38.3–68.2)	92.7 (86.0–96.3)	90.9 (50.8–98.7)	94.7 (68.1–99.2)	75.3 (56.6–86.8)
Localization									
Supratentorial	86.5 (82.7–89.3)	81.0 (60.2–91.6)	84.9 (78.7–89.4)	58.8 (32.5–77.8)	86.1 (67.0–94.6)	93.9 (87.7–97.1)	81.8 (44.7–95.1)	94.1 (65.0–99.2)	84.1 (62.9–93.7)
Infratentorial	70.0 (65.2–74.4)	57.6 (37.0–73.6)	86.9 (80.6–91.3)	58.4 (49.3–66.5)	38.6 (21.3–55.6)	79.6 (39.3–94.5)	100	100	55.2 (32.8–72.9)
Period									
1998–2008	73.0 (68.9–76.7)	63.0 (47.5–75.2)	79.4 (73.0–84.4)	60.0 (49.1–69.3)	62.5 (40.3–78.4)	85.7 (76.2–91.6)	87.5 (38.7–91.1)	86.7 (56.4–96.5)	50.0 (31.9–65.7)
2009–2019	83.2 (79.2–86.4)	82.3 (60.1–93.2)	88.7 (83.1–92.6)	58.4 (44.6–69.9)	60.3 (42.2–74.4)	98.2 (87.8–99.7)	85.7 (33.4–97.9)	100	81.3 (60.6–91.8)

Abbreviations: 5‐y OS, Five‐year overall survival; Infratentorial: cerebellum, brain stem; Supratentorial: cerebrum, supratentorial central area, hypothalamic or pituitary region, optic nerve, pineal gland.

When stratifying the 5‐year OS on age at diagnosis, survival improved in children aged 0–4 years from 68.5% (95% CI 61.2–74.7) in 1997–2008 to 83.6% (95% CI 76.8–88.6) in 2009–2019 (*p* = 0.0006). The 5‐year OS also increased from 75.7 (95% CI 70.6–80.1) in 1997–2008 to 81.5% (95% CI 76.0–85.8) in 2009–2019 in children aged 5–14 years, although the increase was not significant (*p* = 0.16) (Figure 2). Table 3 describes 5‐year overall survival of ICCC‐3 subgroups according to age at diagnosis, gender, localization, and time period.

The 5‐year OS varied considerably according to the histology of CNS tumor. The histological type with the highest 5‐year OS was intracranial and intraspinal germ cell tumors (93.2%, 95% CI 75.1–98.3), while embryonal tumors (59.3%, 95% CI 50.9–66.7) had the poorest survival (Figure S2). Furthermore, glioblastoma had a particularly poor prognosis, and only a minority of these children (6/23) were alive at the end of the study period.

The risk of death according to univariate and multivariate analyses of gender, age group, tumor localization, and time period is presented in Table 4. A statistically significant reduction of the risk of death was observed in children diagnosed with tumors in 2009–2019 compared to 1997–2008 and in supratentorial tumors compared to infratentorial tumors.

**TABLE 4:** Hazard ratios of risk of death according to gender, age, localization, and time period evaluated by univariate and multivariate analyses using Cox proprtional hazards model

	Univariate	Multivariate
Gender		
Boys	Ref	Ref
Girls	1.09 (0.84–1.41)	1.13 (0.88–1.47)
Age		
0–4 years	Ref	Ref
5–14 years	0.88 (0.68–1.15)	0.89 (0.68–1.16)
Localization		
Supratentorial	Ref	Ref
Infratentorial	2.14 (1.60–2.88)	2.16 (1.61–2.90)
Period		
1998–2008	Ref	Ref
2009–2019	0.62 (0.47–0.82)	0.63 (0.47–0.83)

## 4 DISCUSSION

This comprehensive and up‐to‐date study of incidence, survival, and treatment data accurately reflects the incidence, survival, and primary treatment of CNS tumors diagnosed in children aged less than 15 years in Denmark in the period 1997–2019. Based on almost complete national register data, 949 tumors were identified in the 23‐year study period. We included both benign and malignant tumors according to the International Classification of Childhood Cancer main group III and subgroup Xa^7^ as well as treatment information.

### 4.1 Incidence

We found an annual ASR of 42.1 per million person‐years, which is broadly similar to results reported for the same time period from other high‐income countries such as France, the United Kingdom, Australia, Canada, and the United States.^2^, ^11^, ^12^, ^13^, ^21^ The incidence was stable between 1997 and 2019 with only minor fluctuations. High survival and incidence rates are positively associated with the national per capita income level^22^, ^23^ and are probably related to better diagnostic facilities and access to treatment.

An increased incidence of CNS tumors has been reported in both Denmark and worldwide throughout the mid‐1980s as new diagnostic technologies were implemented and the use of MRI became widespread;^8^, ^24^ however, a relatively stable incidence has been reported in Denmark in children aged 0–19 years from 1977 to 2014.^10^ We included children 0–14 years, and our finding that incidence rates were stable after 2000 is in line with other studies from Denmark and Europe.^4^, ^10^, ^12^, ^13^ Other register‐based studies have reported an increased overall incidence in Europe and especially in Western Europe.^3^ Not all studies are complete in their registration and may differ in registration practices, which previously has been addressed,^25^ thus contributing to the complexity of international comparison.

The most common histological type, astrocytoma, accounted for 40% of all CNS tumors in our study, whereas the most frequent single histological type was pilocytic astrocytoma, accounting for 52% of astrocytomas and 21% of all histological types, which is similar to results from previous studies.^11^ As pilocytic astrocytoma accounts for 21% of all histological types in this study, future analyses may show an artificial increase in the proportion of benign compared to malignant tumors.

We provided incidence rates not only of the most frequent long‐established histological types, but also of newer entities in the third edition of the ICD‐O such as dysembryoblastic neuroepithelial tumor (DNET) and ATRT.^17^ DNET is a low‐grade glio‐neuronal tumor with an incidence similar to ganglioglioma, accounting for 43% of mixed glial‐neuronal tumors in our study, which is comparable to other studies.^11^ ATRT (9408/3) is an aggressive histological type and the second most common embryonal tumor after medulloblastoma. As ATRT often presents in the first 2 years of life, radiation treatment is commonly withheld to protect the immature brain. Due to improved diagnostics, ATRT became an independent entity in the same ICCC‐3 subgroup after the year 2000. Previously, ATRT could be interpreted as medulloblastoma when located in the cerebellum in children aged 0–2 years.^6^, ^26^

As new entities arise, others are reclassified, as illustrated by the continuous updates of the WHO classification.^27^ Primitive neuroectodermal tumors of the CNS (CNS‐PNET) are highly malignant neoplasms, but neuropathologic diagnosis has been challenging due to a lack of defining molecular markers and a histologic overlap with other high‐grade neuroepithelial tumors.

In contrast to other population‐based studies on incidence rates, we also provided information on treatment. Many tumors are of benign origin, and more than one‐third of the included tumors (39%) were treated with surgery alone without chemotherapy and radiation, whereas malignant tumors require multimodal treatment.^28^ Childhood CNS tumors constitute a highly heterogeneous group, where different treatment modalities are used. Radiation therapy is preferably postponed or avoided in children younger than 4 years due to potential side effects such as cognitive impairment. Chemotherapy is preferred in young children with malignant, unresectable, or recurrent tumors.

Children were offered proton therapy abroad, if found beneficial. In Denmark, proton therapy became available for children in 2019.

### 4.2 Survival

Overall 5‐year survival rates for Danish children with CNS tumors were similar to those reported in France,^12^ Sweden,^6^ and the United States,^29^ but survival rates can be difficult to compare because selection criteria differ. Data from the United States were based on SEER Statistics for children 0–19 years of age, and a 5‐year survival rate including benign tumors from 2010 to 2016 of 74.9% was reported. In our study, 5‐year overall survival rate improved from 73.0% in 1997–2008 to 83.2% in 2009–2019. The improved overall survival was statistically significant in children aged 0–4 years, where the 5‐year overall survival increased from 68.5% in 1997–2008 to 83.6% in 2009–2019. In the years prior to our study period, a Danish study reported a remarkable improvement of the 10‐year survival in Danish children aged 5–14 years. The survival increased from 59% in 1980–1987 to 74% in 1988–1996,^5^ which probably explains why we did not find a significant increase in 5‐year overall survival for children aged 5–14 years in our study.

Over the last two decades, cancer survival has increased remarkably due to advances in molecular characterization of the tumors^30^ as well as improved surgery, chemotherapy, and radiation protocols in Denmark and in other countries.^14^ Although the 10‐year survival exceeded 70% in our study, this number included 39% of benign tumors treated with surgery alone. It is well known that survival differs considerably between histological types.^31^ Malignant CNS tumors such as embryonal tumors or glioblastoma still have a poor prognosis, and the OS of these tumors continued to decline 5–10 years after diagnosis. Future perspectives on how to increase survival of children with malignant tumors must include studies of genetic profiling and how genetic profiling could lead to better treatment regimens.

Finally, our analyses showed that supratentorial tumors or tumors diagnosed in the more recent time period had more favorable prognostic factors. Younger children have been reported to have poorer survival in other studies,^6^, ^12^, ^32^ but we were not able to show any difference in survival by either age group or gender. This might be due to the fact that malignant tumors are often located in fossa posterior combined with better resection possibilities in the supratentorial area. However, our results should be interpreted with caution, as our study lacks important clinical data such as tumor grade, which probably has an impact on survival.

### 4.3 Strengths and limitations

Major strengths of this study were the long study period and the high‐quality data based on the DCCR with practically complete coverage. The diagnosis and location of the tumor as well as cancer treatment were validated in medical records. We also excluded all non‐CNS tumors originally registered as CNS tumors, yielding precise incidence rates of true CNS tumors.

The classification system of CNS tumors was changed by WHO in 2016^18^ and continuously updated.^33^ It is now based on molecular biology in combination with histological parameters to define several tumor entities; thus WHO formulated a new concept of how diagnoses should be structured in the molecular era. We were not able to reclassify tumors diagnosed before 2016 to WHO 2016 classification scheme. To use the new classification system retrospectively we would require new immune and next‐generation sequencing evaluations to prevent misclassification.^18^ Genetic profiling of selected tumor biopsies has already proven that diagnoses can be changed.^34^ In the future, with genetic profiling of every single tumor biopsy, the histological distribution may thus be altered.^35^, ^36^ Furthermore, it is important to improve the quality of data collected in registries by adding molecular information and by having uniform complete registration according to recommendations for coding central nervous system tumors.^37^

In conclusion, the present study confirms the results from other recent publications that have reported an incidence of childhood CNS tumors of close to 40 per million person‐years. Incidence rates are stable but remain among the highest in the world. Survival has improved especially among younger children with CNS tumors. Still, some histological types have a particularly poor prognosis.

## CONFLICT OF INTEREST

The authors declare no conflict of interest.

## ETHICAL APPROVEMENT AND CONSENT TO PARTICIPATE

The study was ethically approved by the Central Denmark Region Committee on Health Ethics (file number: 1–10–72–65–19) and relevant data protection agency (Central Denmark Region, file number: 1–16–02–109–19). The study was performed in accordance with the Declaration of Helsinki.

## Supporting information

Fig S1

Fig S2

Table S1‐S4

## Data Availability

The study was ethically approved by the Central Denmark Region Committee on Health Ethics (file number: 1–10–72–65–19) and relevant data protection agency (Central Denmark Region, file number: 1–16–02–109–19). The study was performed in accordance to the Declaration of Helsinki.
