# Supplementary figures and images for: Incidence and survival of childhood central nervous system tumors in Denmark, 1997–2019
Source: Cancer Med. 2021 Nov 19;11(1):245–56. doi: 10.1002/cam4.4429 (PMC8704152; doi:10.1002/cam4.4429)

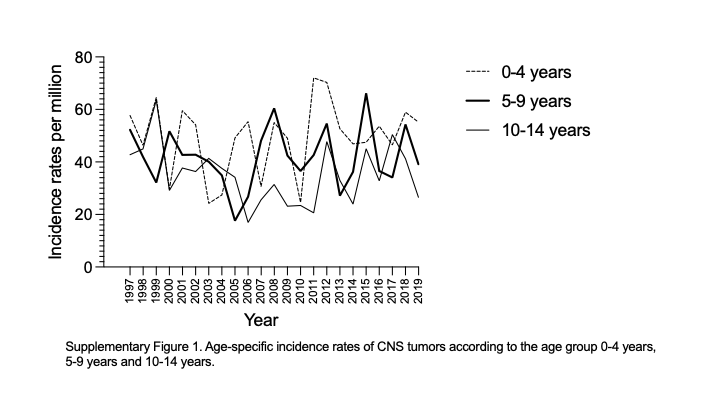

Supplement: Supplementary file 1 — Fig S1 [file CAM4-11-245-s002.tiff]

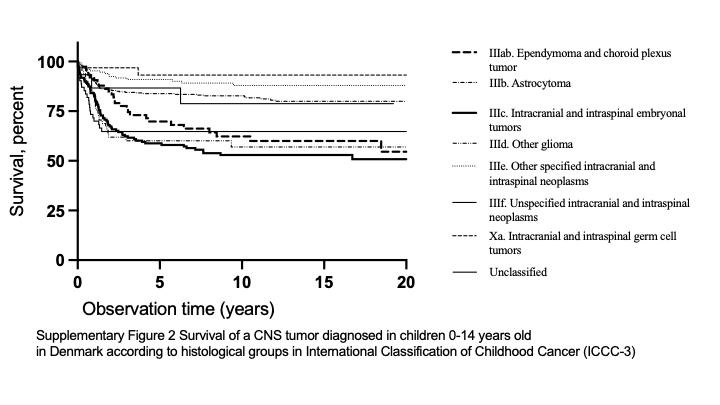

Supplement: Supplementary file 2 — Fig S2 [file CAM4-11-245-s003.tiff]
