# Supplementary material for: Incidence and survival of childhood central nervous system tumors in Denmark, 1997–2019
Source: Cancer Med. 2021 Nov 19;11(1):245–56. doi: 10.1002/cam4.4429 (PMC8704152; doi:10.1002/cam4.4429)
Supplement: Supplementary file 3 — Table S1‐S4 [file CAM4-11-245-s001.doc]

| \| Supplementary Table S1. CNS tumors according to ICC3 subgroups and morphology codes based on the International Classification of Diseases for Oncology \| \| \| \| \| --- \| --- \| --- \| --- \| \|  \| *n* \| % of CNS tumors \| Morphology codes ICD-O \| \| All CNS tumors \| 949 \| 100 \|  \| \| IIIa. Ependymomas and choroid plexus tumor \| 77 \| 8.1 \| 9383, 9390–9394 \| \| IIIb. Astrocytomas \| 385 \| 40.6 \| 9380, 9384, 9400–9411, 9420, 9421–9424, 9440–9442 \| \| IIIc. Intracranial and intraspinal embryonal tumors \| 160 \| 16.9 \| 9470–9474, 9480, 9508  9501–9504 \| \| IIId. Other gliomas \| 66 \| 7.0 \| 9380, 9381, 9382, 9430, 9444, 9450, 9451, 9460 \| \| IIIe. Other specified intracranial and intraspinal neoplasms \| 152 \| 16.0 \| 8270–8281, 8300, 9350–9352, 9360–9362, 9412, 9413, 9492, 9493,  9505–9507, 9530–9539, 9582 \| \| IIIf. Unspecified intracranial and intraspinal neoplasms \| 15 \| 1.6 \| 8000–8005 \| \| Xa. Intracranial and intraspinal germ cell tumors \| 32 \| 3.4 \| 9060–9065, 9070–9072, 9080–9085, 9100, 9101 \| | | | | | | | | | | | | | | |
| --- | --- | --- | --- | --- | --- | --- | --- | --- | --- | --- | --- | --- | --- | --- | --- | --- | --- | --- | --- | --- | --- | --- | --- | --- | --- | --- | --- | --- | --- | --- | --- | --- | --- | --- | --- | --- | --- | --- | --- | --- | --- | --- | --- | --- | --- | --- | --- | --- | --- | --- | --- | --- | --- | --- |
| **Supplementary Table S2. Age distribution and treatment characteristics as well as 5- and 10-year survival of selected sub-types of CNS tumors diagnosed in children < 15 years in Denmark from 1997–2019** | | | | | | | | | | | | | | |
| Selected sub-type | 0–4 years, n | % | 5–9 years, n | % | 10–14 years, n | % | Surgery, n | % | Chemotherapy, n | % | Radiation, n | % | 5year survival (95% CI) | 10-year survival  (95% CI) |
| Pilocytic astrocytoma | 75 | 37.5 | 70 | 35.0 | 55 | 27.5 | 192 | 96.0 | 31 | 15.5 | 17 | 8.5 | 96.7 (92.8–98.5) | 95.1 (90.3–97.6) |
| Optic nerve glioma | 36 | 72.0 | 11 | 22.0 | 3 | 6.0 | 10 | 20.0 | 22 | 44.0 | 4 | 8.0 |  |  |
| Glioblastoma | 8 | 34.8 | 7 | 30.4 | 8 | 34.8 | 20 | 87.0 | 16 | 69.6 | 14 | 60.9 |  |  |
| Medulloblastoma | 46 | 37.1 | 54 | 43.5 | 24 | 19.4 | 117 | 94.4 | 105 | 84.7 | 91 | 73.4 | 62.3 (52.6–70.3) | 57.9 (48.1–66.4) |
| Embryonal tumor NOS | 5 | 31.3 | 8 | 50.0 | 3 | 18.8 | 15 | 93.8 | 10 | 62.5 | 9 | 56.3 |  |  |
| Atypical teratoid/rhabdoid tumor | 16 | 84.2 | 2 | 10.5 | 1 | 5.3 | 16 | 84.2 | 15 | 78.9 | 13 | 68.4 |  |  |
| Germinoma | <3 | 5.6 | 11 | 61.1 | 6 | 33.3 | 15 | 83.3 | 17 | 38.9 | 17 | 94.4 | 93.2 (75.1–98.3) | 93.2 (75.1–98.3) |
| Teratoma | 8 | 57.1 | <3 | 14.3 | 4 | 28.6 | 13 | 92.9 | 5 | 35.7 | 4 | 28.6 |  |  |
| Children <24 months | – |  | – |  | – |  | 119 | 81.0 | 65 | 44.2 | 25 | 17.0 |  |  |
| Intraspinal tumor | 14 | 31.1 | 13 | 28.9 | 18 | 0.4 | 41 | 91.1 | 11 | 24.4 | 13 | 28.9 |  |  |

|  | | | | | | | | | | | | | | |
| --- | --- | --- | --- | --- | --- | --- | --- | --- | --- | --- | --- | --- | --- | --- |
| Supplementary Table S3. Incident cases with age-specific incidence rate (ASI) by histology, age and gender distribution of tumors diagnosed in Denmark in children < 15 years from 1997–2019 | | | | | | | | | | | | | | |
| Histological type | Both genders (*n*) | ASI | ASI for boys | ASI for girls |  | Both genders (*n*) | ASI | ASI for boys | ASI for girls |  | Both genders (*n*) | ASI | ASI for boys | ASI for girls |
|  | 0–4 years | | | |  | 5–9 years | | | |  | 10–14 years | | | |
| All CNS Tumors (adjusted for year of diagnosis) | 363 | 47.7 | 48.5 | 49.7 |  | 320 | 40.7 | 43.3 | 40.2 |  | 266 | 34.7 | 32.6 | 37.6 |
| IIIab. Ependymoma and choroid plexus tumor | 46 | 6.2 | 6.9 | 5.6 |  | 16 | 2.1 | 1.8 | 2.4 |  | 15 | 2.0 | 2.3 | 1.6 |
| IIIb. Astrocytoma | 153 | 20.7 | 17.1 | 24.4 |  | 129 | 16.8 | 17.1 | 16.6 |  | 102 | 13.4 | 10.0 | 16.9 |
| IIIc. Intracranial and intraspinal embryonal tumors | 68 | 9.2 | 10.0 | 8.3 |  | 64 | 8.3 | 8.9 | 7.8 |  | 28 | 3.7 | 3.8 | 3.5 |
| IIId. Other glioma | 21 | 2.8 | 2.9 | 2.8 |  | 26 | 3.4 | 3.8 | 2.9 |  | 19 | 2.5 | 2.3 | 2.7 |
| IIIe. Other specified intracranial and intraspinal neoplasms | 34 | 4.6 | 5.5 | 3.6 |  | 52 | 6.8 | 7.4 | 6.2 |  | 66 | 8.7 | 9.0 | 8.3 |
| IIIf. Unspecified intracranial and intraspinal neoplasms | 4 | 0.5 | 1.1 | 0 |  | 6 | 0.8 | 1.0 | 0.5 |  | 5 | 0.7 | 0.5 | 0.8 |
| Xa. Intracranial and intraspinal germ cell tumors | 9 | 1.2 | 1.1 | 1.4 |  | 8 | 1.0 | 0.8 | 1.3 |  | 15 | 2.0 | 2.3 | 1.6 |
| Unclassified | 28 | 3.8 | 4.0 | 3.6 |  | 19 | 2.5 | 2.5 | 2.4 |  | 16 | 2.1 | 2.3 | 1.9 |

| Supplementary Table S4. Tumor topography by age groups and age-specific incidence rates (ASI) expressed per million person-years of CNS tumors diagnosed in Denmark in children < 15 years from 1997-2019 | | | | | | | | | | | | | | | | |
| --- | --- | --- | --- | --- | --- | --- | --- | --- | --- | --- | --- | --- | --- | --- | --- | --- |
| Tumor topography | ASI | Both genders, *n* | Boys, n | Girls, n |  | ASI | Both genders, *n* | Boys, n | Girls, n |  | ASI | Both genders, *n* | Boys, n | Girls, n | Total, n |  |
|  | 0–4 years | | | |  | 5–9 years | | | |  |  | 10–14 years | | |  |  |
| Cerebellum | 19.6 | 145 | 78 | 67 |  | 16.6 | 127 | 73 | 54 |  | 9.2 | 70 | 33 | 37 | 342 |  |
| Cerebrum | 7.7 | 57 | 34 | 23 |  | 6.4 | 49 | 24 | 25 |  | 9.6 | 73 | 32 | 41 | 179 |  |
| Supratentorial central area | 5.1 | 38 | 19 | 19 |  | 3.1 | 24 | 12 | 12 |  | 3.5 | 27 | 12 | 15 | 89 |  |
| Hypothalamus and pituitary | 2.7 | 20 | 10 | 10 |  | 4.6 | 35 | 17 | 18 |  | 3.8 | 29 | 16 | 13 | 84 |  |
| Brain stem | 3.0 | 22 | 8 | 14 |  | 4.4 | 34 | 17 | 17 |  | 2.2 | 17 | 6 | 11 | 73 |  |
| Optic nerve or chiasma | 5.7 | 42 | 20 | 22 |  | 2.4 | 18 | 5 | 13 |  | 0.7 | 5 | <3 | 3 | 65 |  |
| Medulla spinalis | 1.9 | 14 | 7 | 7 |  | 1.7 | 13 | 8 | 5 |  | 2.4 | 18 | 8 | 10 | 45 |  |
| Pineal gland | 0.7 | 5 | <3 | 4 |  | 0.4 | 3 | 3 | <3 |  | 1.8 | 14 | 9 | 5 | 22 |  |
| Unclassified | 2.7 | 20 | 9 | 11 |  | 2.2 | 17 | 9 | 8 |  | 1.7 | 13 | 9 | 4 | 50 |  |
